# Risk factors for diagnosis and treatment delay among patients with multidrug-resistant tuberculosis in Hunan Province, China

**DOI:** 10.1186/s12879-024-09036-2

**Published:** 2024-02-02

**Authors:** Temesgen Yihunie Akalu, Archie C. A. Clements, Eyob Alemayehu Gebreyohannes, Zuhui Xu, Liqiong Bai, Kefyalew Addis Alene

**Affiliations:** 1 School of Population Health, Faculty of Health Sciences, Curtin University, Perth, WA 6102 Australia; 2 Geospatial and Tuberculosis Research Team, Telethon Kids Institute, Perth, WA Australia; 3 Institute of Public Health, College of Medicine and Health Sciences, University of Gondar, Gondar, Ethiopia; 4 Peninsula Medical School, University of Plymouth, Plymouth, UK; 5 School of Allied Health, University of Western Australia, Perth, WA Australia; 6 Xiangya School of Public Health, Central South University, Changsha, China; 7 TB Control Institute of Hunan Province, Changsha, China

**Keywords:** Multidrug-resistant tuberculosis, Diagnosis delay, Treatment delay, Hunan Province, China

## Abstract

**Background:**

Multidrug-resistant tuberculosis (MDR-TB) is a global health threat associated with high morbidity and mortality rates. Diagnosis and treatment delays are associated with poor treatment outcomes in patients with MDR-TB. However, the risk factors associated with these delays are not robustly investigated, particularly in high TB burden countries such as China. Therefore, this study aimed to measure the length of diagnosis and treatment delays and identify their risk factors among patients with MDR-TB in Hunan province.

**Methods:**

A retrospective cohort study was conducted using MDR-TB data from Hunan province between 2013 and 2018. The main outcomes of the study were diagnosis and treatment delay, defined as more than 14 days from the date of symptom to diagnosis confirmation (i.e., diagnosis delay) and from diagnosis to treatment commencement (i.e., treatment delay). A multivariable logistic regression model was fitted, and an adjusted odds ratio (AOR) with a 95% confidence interval (CI) was used to identify factors associated with diagnosis and treatment delay.

**Results:**

In total, 1,248 MDR-TB patients were included in this study. The median length of diagnosis delays was 27 days, and treatment delays were one day. The proportion of MDR-TB patients who experienced diagnosis and treatment delay was 62.82% (95% CI: 60.09–65.46) and 30.77% (95% CI: 28.27–33.39), respectively. The odds of experiencing MDR-TB diagnosis delay among patients coming through referral and tracing was reduced by 41% (AOR = 0.59, 95% CI: 0.45–0.76**)** relative to patients identified through consultations due to symptoms. The odds of experiencing diagnosis delay among ≥ 65 years were 65% (AOR = 0.35, 0.14–0.91) lower than under-15 children. The odds of developing treatment delay among foreign nationalities and people from other provinces were double (AOR = 2.00, 95% CI: 1.31–3.06) compared to the local populations. Similarly, the odds of experiencing treatment delay among severely ill patients were nearly 2.5 times higher (AOR = 2.49, 95% CI: 1.41–4.42) compared to patients who were not severely ill. On the other hand, previously treated TB cases had nearly 40% (AOR = 0.59, 95% CI: 0.42–0.85) lower odds of developing treatment delay compared with new MDR-TB cases. Similarly, other ethnic minority groups had nearly 40% (AOR = 0.57, 95% CI: 0.34–0.96) lower odds of experiencing treatment delay than the Han majority.

**Conclusions:**

Many MDR-TB patients experience long diagnosis and treatment delays in Hunan province. Strengthening active case detection can significantly reduce diagnosis delays among MDR-TB patients. Moreover, giving attention to patients who are new to MDR-TB treatment, are severely ill, or are from areas outside Hunan province will potentially reduce the burden of treatment delay among MDR-TB patients.

**Supplementary Information:**

The online version contains supplementary material available at 10.1186/s12879-024-09036-2.

## Background

Antimicrobial drug resistance is a human-made threat that results from either inadequate treatment, sub-optimal adherence to treatment regimens, or the persistence of resistant strains because of diagnosis or treatment delay [1]. Multidrug-resistant tuberculosis (MDR-TB), defined as tuberculosis (TB) resistant to at least isoniazid and rifampicin [2], is a major threat to global health [3]. MDR-TB is more difficult to diagnose and treat than drug-susceptible tuberculosis (DS-TB) and is associated with higher treatment costs, longer treatment durations, and poorer treatment outcomes [2]. Globally, nearly half a million people developed MDR-TB in 2021, with only 32% of these patients receiving treatment [2].

China has the second-highest MDR-TB burden, accounting for 14% of the global burden share [4]. China aims to achieve the World Health Organization (WHO) 2021 report END-TB strategy targets to reduce TB incidence by 90%, reduce TB mortality by 95%, and eliminate catastrophic costs in TB-affected households by 2035 [5]. China has implemented phenotypic and molecular diagnosis tests, including Gene-Xpert MTB/RIF, to minimize diagnosis delay, which may help achieve these targets. Moreover, China’s health system adopted the Directly Observed Treatment Short course approach to improve MDR-TB treatment success rates [6], but its implementation is poor [7].

Early diagnosis and initiation of MDR-TB treatment are crucial for achieving the global END-TB strategy targets by reducing MDR-TB infection and improving favourable treatment outcomes [8]. Delays in diagnosis and treatment of MDR-TB are associated with increased risk of morbidity [9], mortality, and community transmission, as well as increased treatment costs [10]. Recently, the introduction of the Gene-Xpert MTB/RIF Assay has improved the early detection of TB, MDR-, and extensively drug-resistant (XDR)-TB.

Studies among patients with DS-TB have reported that sociodemographic, behavioural, and clinical factors, including attitudes, knowledge, occupation, and educational status of patients, the presence of diabetes mellitus, and access to TB treatment centres, affect timely diagnosis and commencement of TB treatment [11]. However, the risk factors associated with diagnosis and treatment delays in patients with MDR-TB are yet to be robustly investigated, including in high-burden countries such as China. Understanding the factors that influence diagnosis and treatment delays will assist clinicians in identifying patients at higher risk of diagnosis and treatment delay, increasing the chance of timely diagnosis and treatment and good MDR-TB treatment outcomes, and reducing the chance of onward transmission. Therefore, this study aimed to evaluate diagnosis and treatment delays and risk factors for these delays among MDR-TB patients in Hunan province, China.

## Methods

### Study area

A retrospective cohort study was conducted in Hunan province, China. Hunan is one of the 34 provincial regions in China. It has an estimated total area of 211,800 square kilometres’ (81,800 square miles) and has just over 66 million inhabitants as of 2020. According to the census conducted in 2022, Hunan province has 41 ethnic groups. Of these, 89.79% identified as Han, and the remaining 10.21% as one of the minority groups, including Tujia, Miao, Dong, Yao, Bai, Hui, Zhuang, Uyghurs, and so on [12]. The proportion of TB cases that are MDR-TB in Hunan province ranges from 10.6% to 25.2%, which is significantly higher than the national average of 1.8% [13]. Hunan Chest Hospital is the only chest hospital in Changsha (the province’s capital) that provides diagnosis and treatment services for patients with MDR and Extensively Drug-resistant (XDR)-TB. The hospital commenced MDR- and XDR-TB care in 2011 and serves as a referral hospital for presumptive DR-TB patients.

### Data sources and variables

The study population was all MDR-TB and XDR-TB patients enrolled at Hunan Chest Hospital between 2013 and 2018. Data were obtained from an internet-based TB management system administered by the TB Control Institute of Hunan province. All pulmonary or extrapulmonary MDR- and XDR-TB patients enrolled during the study period were included. The data contained demographic variables (age, gender, year of diagnosis, occupational status, residence, ethnicity, patient source, and detainee status) and clinical factors (initial date of symptoms, date of diagnosis, date of treatment commencement, registration type, diagnosis type, diagnosis institution type, and treatment delay).

### Definition of variables

Multidrug-resistant tuberculosis (MDR-TB) is defined as a TB patient who is resistant to at least isoniazid and rifampicin. Extensively drug-resistant (XDR)-TB is defined as a TB patient resistant to isoniazid, rifampicin, fluoroquinolones, and either bedaquiline or linezolid (or both) [14]. We defined a total interval as the entire time required from the onset of symptoms to the start of MDR-TB treatment, which was divided into diagnosis and treatment intervals [15]. Diagnosis interval was defined as the time from the onset of symptoms to the date of MDR-TB confirmation [11]. Treatment delay was defined as the time from MDR-TB confirmation to commencement of MDR-TB treatment [16]. Severely ill was defined as a patient with severe comorbidities, having persistent coughing with breathlessness and weakness with extreme weight loss. Hunan ethnic classification recognizes 55 minority groups, including the Han majority.

### MDR- TB diagnosis in Hunan province

Hunan Chest Hospital follows WHO-recommended methods for the diagnosis and treatment of MDR-TB. Clinical assessments based on symptoms, microscopic sputum examinations, radiological examinations, and molecular techniques such as Line Probe Assay and Gene Xpert are commonly used to diagnose MDR-TB in the province.

There are 131 counties in Hunan province, and only 32 counties can provide comprehensive diagnosis services, including culture. In Hunan Chest Hospital, drug susceptibility testing (DST) is mainly carried out to diagnose MDR-TB. As a result, sputum specimens from all culture-positive TB patients from all parts of the province are referred to the Hunan Chest Hospital for DST. In the hospital, phenotypic DST based on solid and liquid culture techniques and molecular methods using line probe assays and Xpert® MTB/RIF are performed. The Hunan Chest Hospital performs DST for rifampicin, isoniazid, ethambutol, streptomycin, kanamycin, and ofloxacin. Solid and liquid cultures are used to follow up on patients’ progress and treatment outcomes.

### Data analysis

Data in an Excel spreadsheet was translated from Mandarin to English and exported to Stata version 17 software for analysis. Descriptive statistics were conducted and presented as frequencies (percentages) for categorical variables and medians with interquartile ranges (IQR) for continuous variables. The outcome variables (diagnosis and treatment intervals) were calculated in days and categorized as delayed and non-delayed. The number of days between the onset of symptoms and diagnosis confirmation and between diagnosis and commencement of MDR-TB treatment were calculated. A 14-day cut-off point was used to dichotomize diagnosis and treatment delay to be consistent with a previous study (https://www.health.nsw.gov.au/Infectious/controlguideline/Pages/tuberculosis.aspx).

We used chi-square tests to assess associations between outcome and explanatory variables. A univariable logistic regression model was first fitted, and variables with a *p*-value of < 0.2 were entered into a multivariable logistic regression model. An adjusted odds ratio (DST) with a 95% CI was used to determine the statistical significance and strength of associations between risk factors and delays in diagnosis and treatment initiation. As there is no cut-off point to classify delay, sensitivity analysis was conducted using a median value to determine the threshold for delay. We also run two separate analyses based on the type of treatment category (new versus retreatment). A sensitivity analysis was also conducted using a negative binomial regression model, whereby the outcomes were taken as counts. Hosmer–Lemeshow’s goodness of fit test was used to assess model fitness. As the mean and the variance were not equal, the negative binomial model was used over the Poisson regression model for the count data analysis. An adjustive relative risk (ARR) with a corresponding 95% CI was used to declare statistical significance.

## Results

In this study, 1,248 MDR-TB patients were included. The mean age of respondents was 43 years, with an SD of 12 years. The majority were male (73.9%), farmers (79.3%), Han ethnicity (92.0%), and new treatment patients (83.1%) (Table 1).

**Table 1:** Socio-demographic and clinical characteristics of multidrug-resistant tuberculosis patients in Hunan province

Variables	Frequency (%)
**Gender**	
Female	326 (26.1)
Male	922 (73.9)
**Age**	
< 15	26 (2.1)
15–24	174 (13.9)
25–44	277 (22.2)
45–64	506 (40.6)
> = 65	265 (21.2)
**Year of diagnosis**	
2013	44 (3.5)
2014	63 (5.1)
2015	186 (14.9)
2016	301 (24.1)
2017	271 (21.7)
2018	383 (30.7)
**Current address**	
Hong Kong, Macau, and Taiwan	37 (3.0)
From Hunan, but outside Changsha	1,151 (92.2)
From another province of mainland China	53 (4.3)
From Changsha	7 (0.5)
**Occupation**	
Children and students	42 (3.4)
Government employees	23 (1.8)
Farmers	990 (79.3)
Housekeepers	89 (7.1)
Privately employee	19 (1.5)
Retired	58 (4.7)
Others^a^	27 (2.2)
**Ethnicity**	
Han	1,149 (92.0)
Dong	11 (0.9)
Miao	22 (1.8)
Tujia	54 (4.3)
Yao	7 (0.6)
Others^b^	5 (0.40)
**Patient source**	
Health check	11 (0.9)
Recommended for consultation due to symptoms	11 (0.9)
Referral	393 (31.5)
Seeking consultation due to symptoms	385 (30.9)
Identified through contact tracing	448 (35.8)
**Detainees**	
No	1,242 (99.5)
Yes	6 (0.5)
**Treatment category**	
New treatment	1,037 (83.1)
Retreatment	211 (16.9)
**1**^**st**^** diagnosis institution type**	
Communicable disease control	998 (80.7)
General hospital	224 (18.1)
Specialist hospital	1 (0.1)
TB dispensary	14 (1.1)
**Severely ill**	
No	1,197 (95.9)
Yes	51 (4.1)

^a^Housekeeping, housework and unemployed, and known

^b^Bai, Buyi, Dai, Gelao, Hani, Hui, Jingpo, Kazakh, Kirgiz, Korean, Lahu, Li, Lisu, Manchu, Mongolian, Salar, She, Tibetan, Tu, Uighur, Wa, Yi, Zhuang

### Median time to diagnosis and treatment interval among multidrug-resistant tuberculosis patients

Overall, the median diagnosis interval among MDR-TB patients was 27 days (IQR 7–66 days), and the median treatment interval was one day (IQR 0–24 days) (Table 2). The overall prevalence of diagnosis delay among MDR-TB patients was 62.8% (95% CI: 60.1–65.4), and 30.8% (95% CI: 28.3–33.4) of MDR-TB patients experienced treatment delay. The diagnosis and treatment delay distribution are summarized in Supplementary files, Figs. S1 and S2.

**Table 2:** Median diagnosis and treatment delay among multidrug-resistant tuberculosis patients in Hunan province stratified by demographic and clinical characteristics

**Variables**	**Frequency (%)**	**Median time to diagnosis in days** (IQR)	**Median time to treatment in days (IQR)**
**Gender**			
Female	326 (26.1)	30 (8–68)	1 (0–17)
Male	922 (73.9)	26 (6–65)	1 (0–25)
**Year of diagnosis**			
2013	44 (3.5)	23.5 (10–49)	0 (0–3)
2014	63(5.1)	28 (6–65)	1 (0–7)
2015	186 (14.9)	26 (7–77)	1 (0–23)
2016	301 (24.1)	29 (8–62)	1 (0–19)
2017	271 (21.7)	29 (6–70)	1 (0–20)
2018	383 (30.7)	26 (4–65)	3 (0–31)
**Current address**			
Foreign nationality	37 (3.0)	31 (9–75)	10 (10–37)
Hunan province	1,151 (92.2)	26 (6–66)	1 (0–20)
Another province	60 (4.8)	39 (13–64)	8 (0–37)
**Occupation**			
Child and students	42 (3.4)	12.5 (1–32)	4.5 (0–24)
Government employees	23 (1.8)	30 (1–53)	8 (0–19)
Farmers	990 (79.3)	29 (7–67)	1 (0–20)
Housekeepers	89 (7.1)	22 (6–60)	5 (0–26)
Privately employed	19 (1.5)	22 (1–91)	23 (0–61)
Retired	58 (4.7)	24.5 (4–81)	12 (1–38)
Others^a^	27 (2.2)	37 (10–75)	0 (0–37)
**Ethnicity**			
Han	1,149 (92.1)	27 (7–65)	1 (0–26)
Dong	11 (0.9)	44 (21–291)	1 (0–3)
Miao	22 (1.8)	36 (12–96)	3.5 (0–16)
Tujia	54 (4.2)	27.5 (3–77)	1 (0–5)
Yao	7 (0.6)	14 (4–42)	1 (0–3)
Others^b^	5 (0.4)	10 (9–12)	17 (1–50)
**Patient source**			
Health check	11 (0.9)	3 (0–10)	5 (0–18)
Recommended for consultation due to symptoms	11(0.9)	34 (10–60)	0 (0–4)
Referral	393 (31.5)	22 (3–65)	2 (0–16)
Seeking consultation due to symptoms	385 (30.9)	30 (13–69)	0 (0–1)
Identified through contact tracing	440 (35.8)	27.5 (5–66)	19 (0–42.5)
**Detainees**			
No	1,242 (99.5)	27 (7–66)	1 (0–24)
Yes	6 (0.5)	14 (5–35)	13 (0–50)
**Treatment category**			
New treatment	1,037 (83.1)	27 (6–63)	1 (0–26)
Retreatment	211 (16.9)	29 (12–84)	1 (0–11)
**1**^**st**^** diagnosis institution type**			
CDC	998 (80.7)	28 (7–65)	1 (0–26)
Hospital	225 (18.2)	24 (3–69)	0 (0–16)
TB dispensary	14 (1.1)	17 (10–38)	3 (0–13)
**Severely ill**			
No	1,197 (95.9)	27 (7–66)	1 (0–22)
Yes	51 (4.1)	31 (8–66)	16 (0–34)

*CDC* Communicable Disease Control, *IQR* Inter Quartile Range

^a^Housekeeping, housework and unemployed

^b^Bai, Buyi, Dai, Gelao, Hani, Hui, Jingpo, Kazakh, Kirgiz, Korean, Lahu, Li, Lisu, Manchu, Mongolian, Salar, She, Tibetan, Tu, Uighur, Wa, Yi, Zhuang

### The trend in diagnosis and treatment delay among MDR-TB patients

**Fig. 1:**
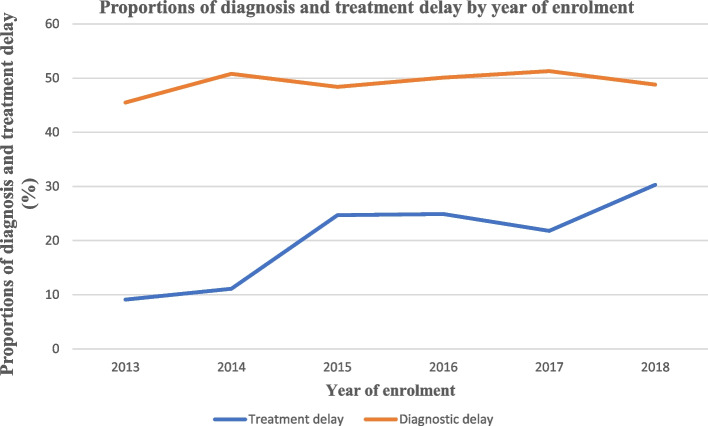
Proportions of diagnosis and treatment delay by year of enrolment

Diagnosis delay among MDR-TB patients increased yearly but not linearly. On the other hand, the treatment delay increased over 2013 and 2015. Then, the trend was steady from 2015 to 2016 and showed a slight reduction between 2016 and 2017. The trend of treatment delay increased alarmingly over a period of 2017 and 2018 (Fig. 1).

### Factors affecting diagnosis delay among patients treated for MDR-TB

The odds of experiencing MDR-TB diagnosis delay among patients who came through referral and tracing was 41% lower (AOR = 0.61, 95% CI: 0.47–0.80) relative to patients identified through consultations due to symptoms. Compared to patients younger than 15 years, patients aged ≥ 65 years had 65% lower odds of experiencing diagnosis delay (AOR = 0.35, 95% CI: 0.14–0.91) (Table 3).

**Table 3:** Univariable and multivariable logistic regression of factors associated with 14 days of multidrug-resistant tuberculosis diagnosis delay

Variables	Diagnosis delay (%)		COR with a 95% CI	*P*-value	AOR with a 95% CI	*P*-value
	**Yes**	**No**				
**Gender**						
Female	214	112	1		1	
Male	570	352	0.84 (0.65–1.10)	0.200	0.80 (0.61–1.05)	0.112
**Age in years**						
< 15	20	6	1		1	
15–64	581	330	0.53 (0.21–1.33)	0.175	0.41 (0.16–1.06)	0.066
≥ 65	183	128	0.43 (0.17–1.09)	0.078	**0.35 (0.14–0.91)**	0.032
**Occupation**						
Students and employed	45	39	1		1	
Farmers	633	357	1.54 (0.60–1.05)	0.060	1.43 (0.90–2.28)	0.128
Others^a^	106	68	1.35 (0.80–2.29	0.262	1.32 (0.77–2.25)	0.315
**Ethnicity**						
Han	724	425	1		1	
Other ethnic minorities	60	39	0.90 (0.98–2.41)	0.195	0.89 (0.58–1.37)	0.184
**Patient source**						
Seeking consultations	279	114	1		1	
Tracing and referral	505	350	0.59 (0.46–0.76)	0.000	**0.59 (0.45–0.76)**	0.000
**Treatment category**						
New treatment	638	399	1		1	
Retreatment	146	65	1.40 (1.02, 1.93)	0.036	1.29 (0.93–1.79)	0.137
**Diagnosis institution**						
CDC	635	365	1		1	
General Hospital	133	92	0.83 (0.62–1.11)	0.190	0.85 (0.62–1.15)	0.175
TB dispensary	16	9	1.02 (0.44–2.32)	0.969	0.91 (0.39–2.12)	0.878

Other ethnic minorities: Dong, Miao, Tujia, Yao

*CDC* Communicable Disease Control, Hosmer and Lemeshow test (Prob > chi2 = 0.3135), *COR* Crude Odds Ratio, *AOR* Adjusted Odds Ratio, *TB* Tuberculosis. Other^a^ Housekeeping and retired, *AOR* Adjusted Odds Ratio, *COR* Crude Odds Ratio, *CI* Confidence Interval

In sensitivity analysis (i.e., using the median 27 days as a cut-off point instead of 14 days), age, occupation of respondents, and patient source of diagnosis were statistically significant factors associated with treatment delay, suggesting the results are sensitive to the selection of the threshold defining a delay. The odds of experiencing diagnosis delay among elderly MDR-TB patients (≥ 65 years) was 60% lower (AOR = 0.40, 0.17–0.94) than among children under 15 years old. The odds of developing diagnosis delay among MDR-TB patients identified through referral and tracing was 25% lower (AOR = 0.75, 0.59–0.96) compared with MDR-TB patients who came to seek consultations due to having had symptoms (Supplementary file Table S1).

No significant variables were identified in the sensitivity analysis that stratified MDR-TB patients as new or previously treated (Supplementary file Table S2).

Supplementary file Table S3 shows the negative binomial regression assessment of factors affecting diagnosis delay among MDR-TB patients. The finding showed that gender, diagnosis institution type, age, and treatment category were significantly associated with diagnosis delay.

### Factors affecting treatment delay among patients treated for MDR-TB

Current residence, treatment category, ethnicity, and severity of illness were associated with treatment delay. The odds of developing treatment delay among foreign nationalities and people from other provinces were almost double (AOR = 1.99, 95% CI: 1.30–3.04) compared to the local populations. The odds of experiencing treatment delay among severely ill patients were 2.58 times higher (AOR = 2.58, 95% CI: 1.46–4.58) than patients who were not severely ill. Previously treated TB cases had 41% lower odds (AOR = 0.59, 95% CI: 0.42–0.85) of treatment delay than new MDR-TB cases. Similarly, other ethnic minority groups had 67% lower odds (AOR = 0.55, 95% CI: 0.33–0.93) of experiencing treatment delay than the Han majority (Table 4).

**Table 4:** Univariable and multivariable logistic regression for treatment delay among multidrug-resistant tuberculosis patients in Hunan Province, 2013–2018

Variables	Treatment delay		COR with 95% CI	*P*-value	AOR with 95%CI	*P*-value
	**Yes**	**No**				
**Gender**						
Female	88	238	1		1	
Male	296	626	1.28 (0.96–1.69)	0.086	1.22 (0.92–1.62)	0.173
**Ethnicity**						
Han	365	784	1		1	
Other minorities	19	80	0.51 (0.30–0.85)	0.010	**0.55 (0.33–0.93)**	0.027
**Treatment category**						
New treatment	337	700	1		1	
Retreatment	47	164	0.60 (0.42–0.84)	0.004	**0.59 (0.42–0.85)**	0.004
**Current residence**						
Local residence	340	811	1		1	
Other provinces and foreign residents	44	53	1.98 (1.30–3.01)	0.001	**1.99 (1.30–3.04)**	0.001
**Severity of illness**						
No	358	839	1		1	
Yes	26	25	2.43 (1.39–4.28)	0.002	**2.58 (1.40–4.41)**	0.001
**Age (in years)**						
< 15	12	14	1		1	
15–64	283	628	0.53 (0.24–1.15)	0.108	0.63 (0.28–1.40)	0.256
≥ 65	222	222	0.47 (0.39–1.85)	0.066	0.55 (0.24–1.25)	0.155
Hosmer–Lemeshow goodness of fit test		**Prob > chi2 = 0.1889**				

*AOR* Adjusted Odds Ratio, *COR* Crude Odds Ratio, *CI* Confidence Interval

A sensitivity analysis was conducted using different thresholds defining a delay, and sensitivity to this threshold was identified. A sensitivity analysis using the third quartile (24 days) showed that only two variables (ethnicity and patient source) remained significant; the odds of experiencing treatment delay among ethnic minority MDR-TB patients were 62% lower (AOR = 0.38, 95% CI: 0.19–0.74) compared to the Han majority. The odds of experiencing treatment delay among patients identified through referral and tracing was 7.39 (4.75–11.49) times higher than patients who came to seek consultations (Supplementary file Table S4).

In a sensitivity analysis based on treatment category (new vs re-treatment), other occupations had 38% higher odds (AOR = 1.38, 95% CI: 1.01–1.90) of experiencing treatment delay than farmers (Supplementary file Table S5).

Findings from the negative binomial model showed that patient source was significantly associated with the risk of treatment delay (Supplementary file Table S6).

## Discussion

This study evaluated risk factors of diagnosis and treatment delay among MDR-TB patients in Hunan province. About 2/3 and 1/3 MDR-TB patients experienced diagnosis and treatment delays, respectively, using a 14-day threshold to define a delay. The median diagnosis and treatment intervals among MDR-TB patients were 27 and one day, respectively. Elderly patients (≥ 65 years) and patients identified through tracing and referral had lower odds of diagnosis delay than their counterparts. Patients with Han ethnicity, previous TB treatment history, residence outside Hunan province, and who were severely ill had a significantly higher probability of experiencing MDR-TB treatment delay.

### Diagnosis and treatment interval for MDR-TB

The median diagnosis interval found in this study was longer than the nine days reported in Myanmar [17] and five days in Bangladesh [18]. The difference could result from using different diagnosis modalities and algorithms to diagnose MDR-TB. On the other hand, the interval was shorter than previously reported in China, with a median diagnosis of 84 days [19]. The difference could be because of the introduction of Gene-Expert, which reduced laboratory result turnaround time in diagnosing MDR-TB patients [20]. Previous studies also showed that using GeneXpert had significantly reduced treatment delay among DR-TB patients [21, 22]. The study also revealed that 2/3 of MDR-TB patients developed diagnosis delays. However, our finding was higher than the WHO recommendations that every patient should have an early diagnosis of TB, including universal Drug Susceptibility Testing (DST) [2]. The WHO recommends that all DR-TB patients need to commence their treatment as soon as possible to prevent unnecessary side effects, complications, and poor treatment outcomes.

The trend of treatment delay increased alarmingly over a period of 2017 and 2018. A possible justification could be the high case burden of MDR-TB in Hunan province between 2017 and 2018, and patients may be obliged to wait longer to commence their treatment. Moreover, study subjects’ variation in terms of MDR-TB signs and symptoms and poor health-seeking behaviour may contribute to the difference. Also, previous studies suggested that the initiation of MDR-TB treatment mainly depends on baseline laboratory investigations and PMDT panel team decisions on DST test results and availability of treatment options [23, 24]. So, the alarming treatment delay in 2017/2018 could be due to the lack of treatment options. However, further research is needed to point out possible factors contributing to the high burden of treatment delay in 2017 and 2018.

Many MDR-TB patients experienced treatment delays in Hunan province, and it is highly recommended that the initiation of prompt MDR-TB treatment following confirmatory diagnosis is prioritized to achieve the END-TB strategy targets. Early diagnosis and treatment of TB is particularly important to minimize community transmission of the disease, reduce side effects, and improve disease progression treatment outcomes and quality of life of the patients. It can also help minimize the patients’ catastrophic costs [25].

There are few studies previously conducted in China to determine MDR-TB diagnosis and treatment delay. Our study is the first to investigate MDR-TB diagnosis and treatment delays, focusing on Hunan Province (one of the provinces with high MDR-TB burden in China). Our study has incorporated important variables such as ethnic minority and residence that have been missed in the previous studies. The first study conducted in Taizhou, Zhejiang Province, showed that the overall diagnosis and treatment delay was not illustrated. Instead, it primarily determines factors associated with waiting time for DST, pre-attrition, time of waiting for treatment, and treatment outcomes and associated factors. Moreover, it did not report the overall diagnosis delay and associated risk factors. On the other hand, a study conducted in China to determined diagnosis and treatment delay among MDR-TB patients lacked socio-demographic variables such as ethnicity and residence [26]. However, in our study, ethnic minorities and residents outside Hunan Province were identified as risk factors for diagnosis and treatment delay. This will add a piece of literature to the existing knowledge. Moreover, none of the studies conducted a sensitivity analysis, which is valuable for better decision-making and more reliable predictions and highlights areas for improvement. As there is no cut-off point to decide diagnosis and treatment delay, applying a sensitivity analysis to show the result at different points is significant.

### Risk factors of MDR-TB diagnosis delay

Previous studies suggested that elderly patients often struggle to access diagnosis and treatment centres and usually rely on their families to visit health facilities [27]. However, our findings showed that patients ≥ 65 years of age had lower odds of diagnosis delay than patients aged < 15 years. A possible explanation could be that MDR-TB diagnosis in children is impacted by different diagnostic approaches; for example, children may be treated for other respiratory tract infections before diagnosis testing is undertaken, resulting in delayed MDR-TB diagnosis. A previous study revealed that the diagnosis of MDR-TB is bacteriological, and children need a systematic diagnosis approach that leads to a longer time to diagnosis of MDR-TB [28]. Moreover, a lack of awareness that children develop MDR-TB, a perceived inability to diagnose active MDR-TB without TST and CXR, and no international guidance on preventive therapy against MDR-TB could contribute to MDR-TB diagnosis delay in children aged < 15 years [29]. Therefore, improving diagnosis protocols in children could minimize diagnosis delay among MDR-TB patients.

In this study, patients identified through tracing and referral had a lower diagnosis delay than patients identified through symptomatic consultations. Moreover, tracing MDR-TB patients might have a significant contribution to MDR-TB patients visiting the health facility timely due to possible consultation, which is a basic component of support and overcoming major barriers that lead to not disclosing the disease due to the high nature of MDR-TB stigmatization. Also, referral reduces waiting times for primary care and minimizes double investigation as they can rely on their pre-referral work-up. The shorter delay among tracing and referral sub-groups may also be caused by the bias of symptom onset time.

Moreover, our findings agree with previous studies carried out on DS-TB patients. For instance, age and active case findings [30] were significantly associated with diagnosis delay among DS-TB patients. Moreover, other sociodemographic variables (educational status, knowledge of MDR-TB, and personal beliefs and attitudes) [31], using traditional medicines and healers, and limited health service access due to geographical location [32] were significantly associated with diagnosis delay among DS-TB patients.

### Risk factors of MDR-TB treatment delay

Regarding treatment delay, Han ethnicity, patients with previous TB treatment history, residence other than Hunan province, and becoming severely ill were significantly associated with MDR-TB treatment delay. This study found that ethnic minority groups in Hunan province had lower odds of experiencing treatment delay than the Han majority. This could be due to improving educational attainment among ethnic minorities, which is highly prioritized in China [33]. This finding is consistent with a study conducted by Gilmour et al. among DS-TB patients in Hunan province; ethnic minority groups had lower odds of experiencing treatment delay than the Han majority. A systematic review and meta-analysis conducted among DS-TB patients in Ethiopia showed that lower educational level was significantly associated with poor health-seeking behaviour [34]. However, further research is recommended to illuminate why the Han majority groups are at higher odds of experiencing treatment delay than ethnic minority groups.

In this study, previously treated MDR-TB cases had lower odds of experiencing MDR-TB treatment delay. This might be because previously treated cases were more knowledgeable about what to do and aware of the severity of the disease and the risk of developing a resistant form of TB, and they already had relationships with clinicians that expedited their care; they were also less likely to experience diagnosis delay than new MDR-TB patients [34, 35].

Patients from locations other than Hunan province had an increased likelihood of experiencing treatment delay. In China, the hukou system, a household registration system, permits a permanent resident for a single address issued by the government of China to each family member given at birth or by employment in formal sectors [36]. The hukou system allows each family member access to essential public services, education, health services, social benefits, and job recruitment by the governmental or private sectors [37]. As a result, patients from Hunan province will have better access to health care and are less likely to experience treatment delays than patients from outside Hunan province. According to previous reports, a previous history of DS-TB treatment [38] and health system factors (staff shortages, cost of services, drug stockout, and poor health infrastructure) [11] are significantly associated with treatment delay.

### Limitations of the study

The study had several limitations. The absence of a WHO-endorsed, international standard cut-off point to determine diagnosis and treatment delay can significantly impact estimates of the burden of delays and cause misclassification bias when investigating factors associated with the delays. Some important variables that might be associated with diagnosis and treatment delay, such as education status, income level, geographical inaccessibility, knowledge of MDR-TB, and presence of comorbidities (e.g., diabetes mellitus, HIV infection, mental ill health), were not assessed in the current study. Future research should prioritize undertaking prospective studies that measure these variables. Only patients attending the designated health facilities were included in the study. This might have caused selection bias, and findings might not be generalizable to all MDR-TB patients in Hunan province. Recall bias might also have been an issue for patients specifying the exact date of symptom onset. Finally, all sputum specimens were transported to Hunan Chest Hospital for DST might introduce turnaround time that will further influence on the diagnosis delay.

## Conclusion

Long diagnosis and treatment delays still occur for many MDR-TB patients in Hunan province. Under-15 children and patients identified through passive case detection were found to experience a higher probability of diagnosis delay. Ethnic minority groups and patients who were previously treated for MDR-TB had lower odds of treatment delay. On the other hand, the odds of treatment delay in patients coming from areas other than the Hunan province and severely ill patients were high. Giving attention to new MDR-TB patients, severely ill patients and patients from outside Hunan province will potentially reduce the burden of treatment delay among MDR-TB patients.

### Supplementary Information

**Additional file 1: Supplementary file Fig S1.** The distribution of diagnosis delay among multidrug-resistant tuberculosis stratified by number of days in Hunan Province. **Supplementary file Fig S2.** The distribution of treatment delay among multidrug-resistant tuberculosis stratified by the number of days. **Supplementary file Table S1.** A sensitivity analysis: Univariable and multivariable logistic regression for diagnosis delay among multidrug-resistant tuberculosis patients in Hunan Province, 2013-2018 (Using median as a cut-off point). **Supplementary file Table S2.** A sensitivity analysis: Univariable and multivariable logistic regression for diagnosis delay stratified by treatment category among multidrug-resistant tuberculosis patients in Hunan Province, 2013-2018. **Supplementary file Table S3.** A sensitivity analysis: Univariable and multivariable negative binomial regression assessment of factors associated with time from symptoms to diagnosis in multidrug-resistant tuberculosis patients registered in Hunan Province, 2013-2018. **Supplementary file Table S4.** A sensitivity analysis: Univariable and multivariable logistic regression for treatment delay among multidrug-resistant tuberculosis patients in Hunan Province, 2013-2018 (Using the upper quartile as a cut-off point). **Supplementary file Table S5.** A sensitivity analysis: Univariable and multivariable logistic regression for treatment delay stratified by treatment category among multidrug-resistant tuberculosis patients in Hunan Province, 2013-2018. **Supplementary file Table S6.** A sensitivity analysis: Univariable and multivariable negative binomial regression assessment of factors associated with time from diagnosis to treatment commencement in multidrug-resistant tuberculosis patients registered in Hunan Province, 2013-2018.

## Data Availability

Data will be available upon request from the corresponding author.

## Abbreviations

AOR: Adjusted Odds Ratio
ARR: Adjusted Relative Risk
CI: Confidence Interval
DST: Drug Susceptibility Testing
DS-TB: Drug Susceptible Tuberculosis
HIV: Human Immune Deficiency Virus
IQR: Inter Quartile Range
MDR-TB: Multidrug Resistant Tuberculosis
TB: Tuberculosis
WHO: World Health Organization
XDR-TB: Extensively Drug Resistant Tuberculosis
