# Supplementary material for: Risk factors for diagnosis and treatment delay among patients with multidrug-resistant tuberculosis in Hunan Province, China
Source: BMC Infect Dis. 2024 Feb 2;24:159. doi: 10.1186/s12879-024-09036-2 (PMC10835895; doi:10.1186/s12879-024-09036-2)
Supplement: Supplementary file 1 — Additional file 1: Supplementary file Fig S1. The distribution of diagnosis delay among multidrug-resistant tuberculosis stratified by number of days in Hunan Province. Supplementary file Fig S2. The distribution of treatment delay among multidrug-resistant tuberculosis stratified by the number of days. Supplementary file Table S1. A sensitivity analysis: Univariable and multivariable logistic regression for diagnosis delay among multidrug-resistant tuberculosis patients in Hunan Province, 2013-2018 (Using median as a cut-off point). Supplementary file Table S2. A sensitivity analysis: Univariable and multivariable logistic regression for diagnosis delay stratified by treatment category among multidrug-resistant tuberculosis patients in Hunan Province, 2013-2018. Supplementary file Table S3. A sensitivity analysis: Univariable and multivariable negative binomial regression assessment of factors associated with time from symptoms to diagnosis in multidrug-resistant tuberculosis patients registered in Hunan Province, 2013-2018. Supplementary file Table S4. A sensitivity analysis: Univariable and multivariable logistic regression for treatment delay among multidrug-resistant tuberculosis patients in Hunan Province, 2013-2018 (Using the upper quartile as a cut-off point). Supplementary file Table S5. A sensitivity analysis: Univariable and multivariable logistic regression for treatment delay stratified by treatment category among multidrug-resistant tuberculosis patients in Hunan Province, 2013-2018. Supplementary file Table S6. A sensitivity analysis: Univariable and multivariable negative binomial regression assessment of factors associated with time from diagnosis to treatment commencement in multidrug-resistant tuberculosis patients registered in Hunan Province, 2013-2018. [file 12879_2024_9036_MOESM1_ESM.docx]

**G**

0

10

20

30

40

Percentages

0-14

15-30

31-60

61-90

91-180

181-270

271-360

>1

year

Distribution of diagnosis delay stratified by number of days.

**Supplementary file Fig S1:** The distribution of diagnosis delay among multidrug-resistant tuberculosis stratified by number of days in Hunan Province

0

10

20

30

40

Percentages

0

day

1-7

8-14

15-30

31-60

61-90

>90

Distribution of treatment delay stratified by number of days.

0

10

20

30

40

Percentages

0-14

15-30

31-60

61-90

91-180

181-270

271-360

>1

year

**Supplementary file Fig S2:** The distribution of treatment delay among multidrug-resistant tuberculosis stratified by the number of days

**Supplementary file Table S1: A sensitivity analysis: Univariable and multivariable logistic regression for diagnosis delay among multidrug-resistant tuberculosis patients in Hunan Province, 2013-2018 (Using median as a cut-off point)**

| **Variables** | **Diagnosis delay (%)** | | **COR with 95% CI** | **AOR with 95%CI** |
| --- | --- | --- | --- | --- |
|  | **Yes** | **No** |  |  |
| **Age**  <15  15-64  >=65 | 17  459  145 | 9  452  166 | 1  0.54 (0.24-1.22)  0.46 (0.20-1.07) | 1  0.43 (0.19-1.01)  **0.40 (0.17-0.94)** |
| **Gender**  Female  Male | 168  453 | 158  469 | 1  0.91 (0.71-1.17) | 1  0.88 (0.68-1.13) |
| **Occupation**  Students and employees  Farmers  Others | 32  503  86 | 52  487  88 | 1  1.68 (1.06-2.65)  1.59 (0.93-2.70tab) | 1  **1.73 (1.08-2.78)**  1.67 (0.97-2.86) |
| **Ethnicity**  Han  Others | 569  52 | 580  47 | 1  1.13 (0.75-1.70) | 1  1.12 (0.74-1.71) |
| **Patient source**  Seeking consultations  Referral and tracing | 212  409 | 181  446 | 1  0.78 (0.62-0.99) | 1  **0.75 (0.58-0.96)** |
| **1^st^ diagnosis institution type**  CDC  Hospital  TB dispensary | 506  108  **7** | 492  117  18 | 1  0.89 (0.67-1.20)  0.37 (0.16-0.91) | 1  0.89 (0.67-1.20)  **0.34 (0.14-0.83)** |
| **Current residence**  Local residence  Other provinces and foreign residents | 566  55 | 585  42 | 1  1.35 (0.89-2.06) | 1  1.48 (0.96-2.27) |
| **Hosmer and Lemeshow test** | **Prob > chi2 = 0.5104** | | | |

AOR: Adjusted Odds Ratio, CDC: Communicable Disease Control, COR: Crude Odds Ratio, CI; Confidence Interval, TB: Tuberculosis

Others

**Supplementary file Table S2:** **A sensitivity analysis:** **Univariable and multivariable logistic regression for diagnosis delay stratified by treatment category among multidrug-resistant tuberculosis patients in Hunan Province, 2013-2018**

| **Variables** | **Only new MDR-TB cases (n=1,037)** | | **Previously treated MDR-TB cases (n=211)** | |
| --- | --- | --- | --- | --- |
|  | COR with 95% CI | AOR with 95% CI | COR with 95% CI | AOR with 95% CI |
| Gender  Female  Male | 1  0.86 (0.65-1.15) | 1  0.75 (0.37-1.51) | 1  0.75 (0.38-1.51) | 1  0.74 (0.36-1.48) |
| Ethnicity  Han  **Other ethnic minorities** | 1  0.84 (0.61-1.12) | 1  0.83 (0.62-1.13) | 1  0.67 (0.31-1.44) | 1  0.65 (0.27-1.61) |
| Diagnosis institution  CDC  General Hospital | 1  0.84 (0.62-1.14) | 1  0.84(0.62-1.15) | 1  0.97 (0.44-2.11) | 1  1.05 (0.47-2.31) |
| Patient source  Seeking consultations  Tracing and referral | 1  0.74 (0.57-0.96) | 1  0.74(0.57-0.97) | 1  0.82 (0.45-1.47) | 1  0.81 (0.45-1.48) |

AOR: Adjusted Odds Ratio, CDC: Communicable Disease Control, COR: Crude Odds Ratio, CI: Confidence interval, MDR-TB: Multidrug-resistant Tuberculosis

**Supplementary file Table S3:** A sensitivity analysis: Univariable and multivariable negative binomial regression assessment of factors associated with time from symptoms to diagnosis in multidrug-resistant tuberculosis patients registered in Hunan Province, 2013-2018

| **Variables** | **Frequencies (%)** | **CRR with 95% CI** | **ARR with 95% CI** |
| --- | --- | --- | --- |
| **Gender**  Female  Male | 326 (26.12)  922 (73.88) | 1  1.96 (1.59-2.42) | 1  **1.85 (1.50-2.29)** |
| **Patient sources**  Seeking consultations  Referral and tracing | 393 (31.49)  855 (68.51) | 1  0.98 (0.80-1.20) | 1  1.01 (0.83-1.24) |
| **Diagnosis institution**  CDC  General Hospital  TB dispensary | 998 (79.97)  225 (18.03)  25 (2.00) | 1  0.87 (0.68-1.11)  0.49 (0.25-0.96) | 1  0.89 (0.70-1.13)  **0.44 (0.23-0.86)** |
| **Current residence**  Local residence  Foreign nationality and other provinces | 1,151 (92.23)  97 (7.77) | 1  0.98 (0.69-1.40) | 1  0.96 (0.67-1.38) |
| **Age (in years)**  <15  15-64  ≥65 | 26 (2.08)  911 (73.00)  311 (24.92) | 1  2.39 (1.23-4.62)  2.53 (1.28-4.98) | 1  **1.94 (1.01-3.73)**  **2.42(1.24-4.74)** |
| **Ethnicity**  Han  Other minorities | 1,149 (92.07)  99 (7.93) | 1  1.18 (0.83-1.67) | 1  1.23 (0.86-1.77) |
| **Treatment category**  New  Retreatment | 1,037 (83.09)  211 (16.91) | 1  1.71 (1.33-2.19) | 1  **1.68 (1.30-2.16)** |
| **Lnalpha** | | | 1.01 (0.94-1.08) |
| **Alpha** | | | 2.75 (2.57-2.94) |
| **LR test of alpha** | | | Prob >= chibar2 = 0.000 |

ARR: Adjusted Relative Risk, CRR: Crude Relative Risk, CI: Confidence Interval, CDC: Communicable Disease Control, LR: Likelihood Ratio

**Supplementary file Table S4: A sensitivity analysis:** **Univariable and multivariable logistic regression for treatment delay among multidrug-resistant tuberculosis patients in Hunan Province, 2013-2018 (Using the upper quartile as a cut-off point)**

| **Variables** | **Treatment delay (%)** | | **COR with 95% CI** | **AOR with 95%CI** |
| --- | --- | --- | --- | --- |
|  | **Yes** | **No** |  |  |
| **Gender**  Female  Male | 88  296 | 238  626 | 1  1.24 (0.91-1.67) | 1  1.23 (0.90-1.70) |
| **Occupation**  Students and employees  Farmers  Others | 23  231  121 | 61  759  121 | 1  0.81 (0.49-1.33)  1.16 (0.65-2.07) | 1  1.09 (0.0.64-1.0.85)  1.24 (0.68-2.26) |
| **Ethnicity**  Han  Other minorities | 365  19 | 784  80 | 1  0.36 (0.19-0.68) | 1  **0.39 (0.20-0.76)** |
| **Patient source**  Seeking consultations due to symptoms Referral and tracking | 36  348 | 357  507 | 1  0.14 (0.09-0.21) | 1  **7.39 (4.75-11.49)** |
| **1^st^ diagnosis institution type**  CDC  Hospital  TB dispensary | 317  61  6 | 681  164  19 | 1  0.69 (0.48-0.99)  0.54 (0.18-1.59) | 1  0.76 (0.52-1.11)  0.65 (0.21-2.03) |
| **Treatment category**  New treatment  Retreatment | 337  47 | 700  164 | 1  0.58 (0.39-0.85) | 1  0.74 (0.49-1.11) |
| **Severely ill**  No  Yes | 358  26 | 839  25 | 1  1.87 (1.05-3.36) | 1  1.67 (0.89-3.11) |
| **Hosmer and Lemeshow test** | **Prob > chi2 = 0.9515** | | | |

AOR: Adjusted Odds Ratio, COR: Crude Odds Ratio, CI: Confidence Interval, CDC: Communicable Disease Control

**Supplementary file Table S5:** **A sensitivity analysis:** **Univariable and multivariable logistic regression for treatment delay stratified by treatment category among multidrug-resistant tuberculosis patients in Hunan Province, 2013-2018**

| **Variables** | **Only new MDR-TB cases (n=1,037)** | | **Previously treated MDR-TB cases (n=211)** | |
| --- | --- | --- | --- | --- |
|  | **COR with 95% CI** | **AOR with 95% CI** | **COR with 95% CI** | **AOR with 95% CI** |
| **Patient source**  Seeking consultations  Referral and tracing | 1  6.01 (4.03-8.97) | 1  5.50 (3.67-8.23) | 1  10.73 (4.04-28.52) | 1  11.95 (4.43-32.23) |
| **Current residence**  Local residence  Foreign & other provinces | 1  2.11 (1.34-3.32) | 1  1.53 (0.96-2.43) | 1  1.29 (0.39-4.27) | 1  0.54 (0.16-1.87) |
| **Severely ill**  No  Yes | 1  2.15 (1.14-4.04) | 1  1.90 (0.97-3.71) | * | * |
| **Ethnicity**  Han  Other minorities | ** | ** | 1  0.71 (0.23-2.20) | 1  0.58 (0.17-1.94) |
| **Hosmer and Lemeshow test** | Prob > chi2 = 0.8213 | | Prob > chi2 = 0.7960 | |

N.B * Chi-square assumption not satisfied.

**removed due to multicollinearities

AOR: Adjusted Odds Ratio, COR: Crude Odds Ratio, CI: Confidence Interval, MDR-TB: Multidrug-resistant Tuberculosis

**Supplementary file Table S6:** A sensitivity analysis: Univariable and multivariable negative binomial regression assessment of factors associated with time from diagnosis to treatment commencement in multidrug-resistant tuberculosis patients registered in Hunan Province, 2013-2018

| **Variables** | **Frequencies (%)** | **CRR with 95% CI** | **ARR with 95% CI** |
| --- | --- | --- | --- |
| **Gender**  Female  Male | 326 (26.12)  922 (73.88) | 1  1.39 (1.04-1.87) | 1  1.27 (0.95-1.70) |
| **Patient sources**  Seeking consultations  Referral and tracing | 393 (31.49)  855 (68.51) | 1  3.66 (2.79-4.80) | 1  **3.59 (2.72-4.74)** |
| **Diagnosis institution**  CDC  General Hospital  TB dispensary | 998 (79.97)  225 (18.03)  25 (2.00) | 1  0.83 (0.59-1.17)  0.67 (0.27-1.70) | 1  0.91 (0.65-1.27)  0.80 (0.32-1.97) |
| **Current residence**  Local residence  Foreign nationality and other provinces | 1,151 (92.23)  97 (7.77) | 1  1.25 (0.77-2.02) | 1  0.99 (0.61-1.58) |
| **Age (in years)**  <15  15-64  ≥65 | 26 (2.08)  911 (73.00)  311 (24.92) | 1  0.65 (0.26-1.60)  0.52 (0.21-1.33) | 1  0.87 (0.36-2.09)  0.64 (0.26-1.57) |
| **Ethnicity**  Han  Other minorities | 1,149 (92.07)  99 (7.93) | 1  0.87 (0.54-1.41) | 1  0.85 (0.53-1.35) |
| **Treatment category**  New  Retreatment | 1,037 (83.09)  211 (16.91) | 1  0.81 (0.57-1.14) | 1  0.79 (0.56-1.11) |
| **Severely ill**  No  Yes | 1,197 (95.91)  51 (4.09) | 1  1.41 (0.73-2.71) | 1  1.28 (0.68-2.43) |
| **/lnalpha** | | | 1.60 (1.52-1.69) |
| **Alpha** |  |  | 4.97 (4.55-5.43) |
| **LR test** | | | Prob >= chibar2 = 0.000 |

ARR: Adjusted Relative Risk, CRR: Crude Relative Risk, CI: Confidence Interval, and CDC: Communicable Disease Control
